# HIV Vif protein in docetaxel treatment of breast cancer cells

**DOI:** 10.1080/07853890.2021.1895585

**Published:** 2021-09-28

**Authors:** Pedro Cipriano, Susana Bandarra, João Gonçalves, Ana Clara Ribeiro, Isabel Barahona

**Affiliations:** a Faculdade de Ciências e Tecnologia, Universidade Nova de Lisboa (FCT-UNL), Monte de Caparica, Portugal; b Laboratório de Biologia Molecular, Centro de Investigação Interdisciplinar Egas Moniz (CiiEM), Egas Moniz Cooperativa de Ensino Superior, Caparica, Portugal; c Research Institute for Medicines (iMed.ULisboa), Faculty of Pharmacy, University of Lisbon, Lisbon, Portugal

## Abstract

**Introduction:**

Breast cancer is one of the most frequently diagnosed cancers in the world and is also the leading cause of death in women diagnosed with this disease. Recently, APOBEC3 proteins have been identified as potent mutagenic agents of genomic DNA associated with the onset, progression and treatment resistance of various types of cancer. On the other hand, Vif1 from HIV-1 and Vif2 from HIV-2 are proteins encoded by HIV-1 and HIV-2, respectively, that during viral infection plays a crucial role in the inhibition/degradation of APOBEC3. In this work it was tested the hypothesis that both Vif1 and Vif2 mediated APOBEC3 inhibition will increase the cytotoxicity of docetaxel in a triple-negative breast cancer cell line.

**Materials and methods:**

Breast cancer HCC1806 cell line was used as well as two new derived cell lines, with vif1 and vif2 genes integrated and expressing vif in fusion with Zs Green fluorescent protein, mentioned hereinafter simply as VIF-1 and VIF-2 cells. Cell viability assays were performed by MTT reduction after 24 h and 48 h exposure of cells (HCC1806, VIF-1 and VIF-2) to different concentrations of docetaxel.

**Results:**

Our results in the presence of docetaxel for 24 h have shown that cell viability decreases in all cell lines around 30% or 40%. Moreover, there is no significant differences in cell viability of parental cell line HCC1806 and any of the modified lines (VIF-1 and VIF-2) at any of the docetaxel concentrations tested (*p*-value > .05 – independent sample *t*-test). Additionally, after 48 h exposure to docetaxel, cell survival also decreases significantly to values between 38 and 43% (Figure 1), but again we could not see significant differences in cell viability among HCC1806, VIF-1 and VIF-2 cells treated to any tested concentration of docetaxel. **Discussion and conclusions:** The hypothesis that Vif will enhance triple negative breast cancer cells sensitivity to treatments with docetaxel was not verified. The lack of effect of Vif in cells sensitivity in the presence of docetaxel may be explained by the fact that cell target of docetaxel is Microtubules and Vif proteins do not interfere with Microtubules. Probably, the presence of Vif will only alter sensibility of breast cancer cells when using drugs that affect the DNA, the known target of APOBEC3. Therefore, treatment with drugs independent of DNA seems to be also independent of APOBEC3 levels in cells.

**Figure 1.**
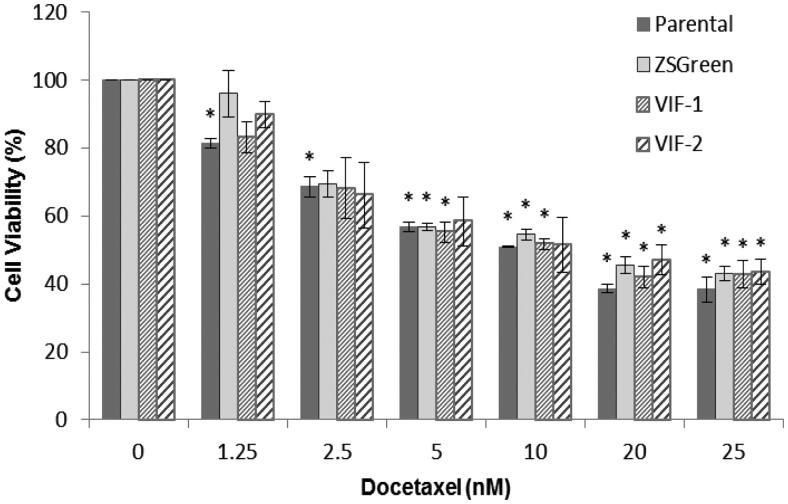
Viability of cells treated with docetaxel for 48 h. Untreated cells are considered to have 100% of viability. Values are present has average ± SEM of three independent MTT assays. *p-value <.05 in relation to untreated cells within the same cell line.

